# Clinical impact of pre-hypertension on the risk of cancer in male and female subjects

**DOI:** 10.1038/s41598-020-66653-y

**Published:** 2020-06-19

**Authors:** Su Hwan Lee, Hye Ah Lee, Sean S. Lee, Seong-Eun Kim, Ki-Nam Shim, Hye-Kyung Jung, Sung-Ae Jung, Jung Hyun Chang, Kihwan Kwon, Wook Bum Pyun, Boyoung Joung, Chang Mo Moon, Junbeom Park

**Affiliations:** 1 Division of Pulmonology, Department of Internal Medicine, Severance Hospital, Yonsei University College of Medicine, Seoul, Republic of Korea; 2 Clinical Trial Center, Ewha Womans University Mokdong Hospital, Ewha Womans University, Seoul, Republic of Korea; 3 Department of Preventive Medicine, College of Medicine, Ewha Womans University, Seoul, Republic of Korea; 4 Program in Liberal Medical Education, The Warren Alpert Medical School, Brown University, Providence, United States; 5 Division of Gastroenterology, Department of Internal Medicine, College of Medicine, Ewha Womans University, Seoul, Republic of Korea; 6 Division of Pulmonary and Critical Care Medicine, Department of Internal Medicine, College of Medicine, Ewha Womans University, Seoul, Republic of Korea; 7 Division of Cardiology, Department of Internal Medicine, College of Medicine, Ewha Womans University, Seoul, Republic of Korea; 8 Department of Cardiology, Yonsei University College of Medicine, Seoul, Republic of Korea; 9 Tissue Injury Defense Research Center, Ewha Womans University, Seoul, Republic of Korea

**Keywords:** Cardiology, Health care, Medical research, Oncology, Risk factors

## Abstract

There are few studies assessing pre-hypertension and an impaired fasting glucose (IFG) and their combined effects on the cancer risk. We investigated the impact of pre-hypertension on cancer risk and IFG, and their combined effects on the cancer risk. This study included 371,762 subjects (≥40 years) who had never been diagnosed with hypertension, diabetes mellitus (DM), and cancer before. During a mean follow-up of 10.06 ± 1.86 years, 35,605 (9.58%) of the subjects developed cancer. In men only, cancer risk was significantly increased with an increase in the blood pressure (BP) (*P* for trend < 0.001), and were increased in the hypertension range, but not the pre-hypertension range. When analyzing the combination effect of BP and fasting glucose, cancer risks were serially increased with an increase in the fasting glucose in a dose-dependent manner, but not with an increase in BP. These results were more consistently significant in the never-smoker and non-alcohol drinking groups. However, in women, there was no significant difference. In conclusions, increased BP status or the fasting serum glucose level status were associated with cancer risk in men. Furthermore, the combination of both pre-hypertension and IFG also was associated with a cancer risk in men.

## Introduction

Approximately 14 million people worldwide are expected to develop cancer annually, and more than 6.2 million are expected to die every year from cancer^1^. Among the many risk factors of cancer, hypertension and diabetic mellitus (DM), which are chronic diseases with high prevalence, are associated with an increased risk of cancer compared to subjects who have a normal blood pressure (BP) and normal fasting serum glucose^2–5^.

In general, pre-hypertension and an impaired fasting glucose (IFG), which are clinically abnormal but do not require medication, are the prior stages to hypertension and DM, respectively^6,7^. However, we previously reported that this pre-hypertension group affected the incidence of cardiovascular diseases, including all-cause mortality and atrial fibrillation^8^. A previous study showed that cancer risk in men showed an incremental increase in BP, even in the pre-hypertensive range. However, that study did not analyze subjects by dividing them into pre-hypertension and hypertension groups^9^. Likewise, the IFG has also been reported to be associated with cancer risk^9–12^. However, some studies did not systematically consider various factors that can affect cancer risk, such as gender, body mass index (BMI), smoking and alcohol habits; or they involved a relatively small study sample^9–15^.

Therefore, we aimed to investigate the impact of pre-hypertension and an IFG on the risk of various cancers in both men and women. Thus, we used the large, comprehensive database of the Korean National Health Insurance Service (NHIS)-Health Screening Cohort to identify the direct association of BP and fasting serum glucose on cancer. Further, all analyses were performed in medication naïve subjects to exclude any little cancerous effect from the medications. Furthermore, we additionally performed a subgroup analysis in subjects without smoking and alcohol intake to exclude the effect of powerful individual-level risk factors that could have an influence on cancer risk^14–16^.

## Methods

### Study design, data source, and study subjects

**Figure 1:**
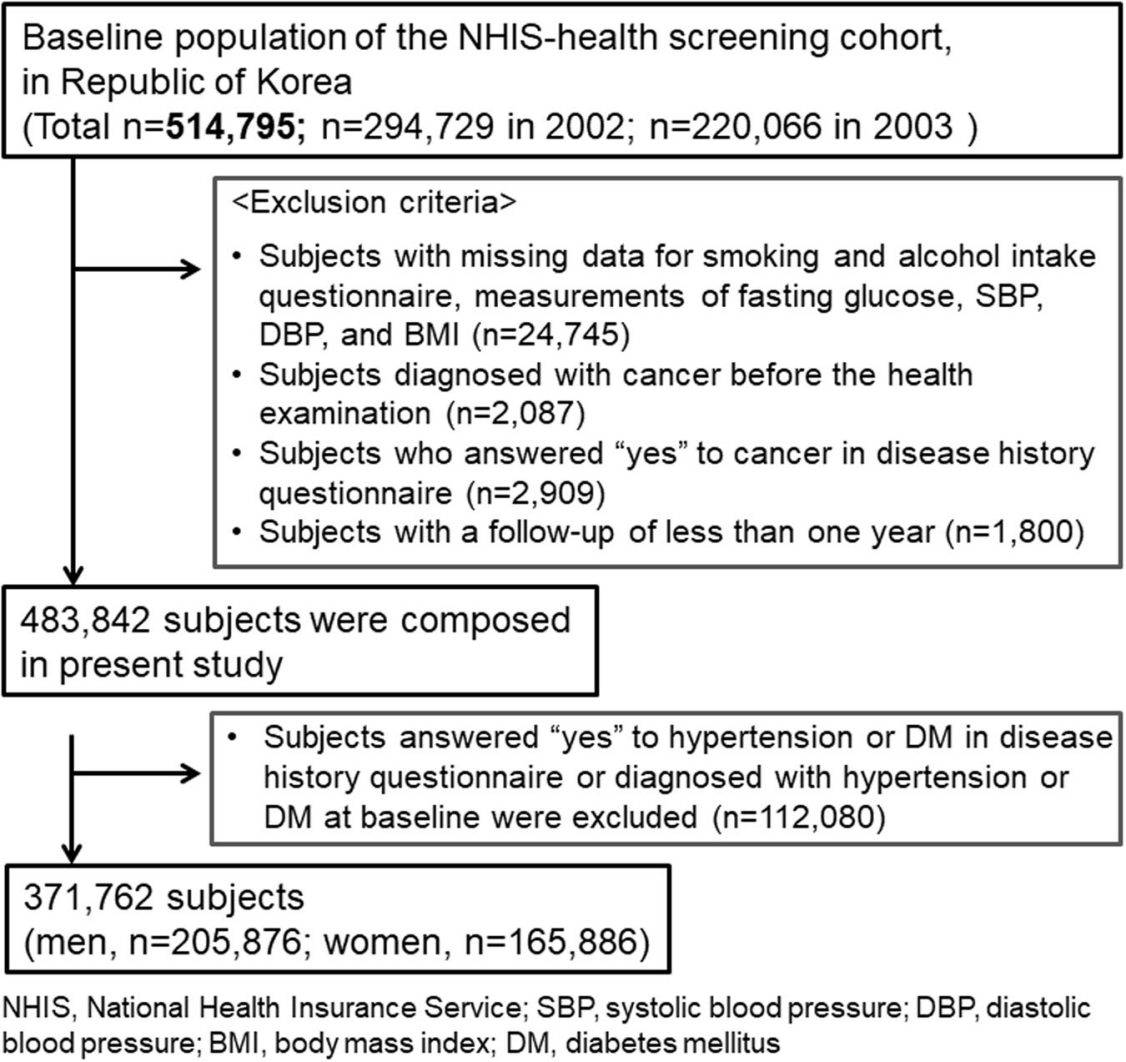
Flow of study.

This study was conducted using data from the NHIS-Health Screening Cohort (NHIS-2018-2-090), which consists of 514,866 participants who were randomly selected from 10% of the eligible Korean population aged 40–79 in 2002 among the national health screening participants during 2002-2003. Detailed information about the NHIS is described in the Supplementary Methods section 1^8,17^. Except for the subjects with an inconsistent date of examination, this study included adults aged 40–79 years (n = 514,795) undergoing National Health screenings at least once between 2002 to 2003. We considered the baseline period of the study as 2002–2003, and the date of the health screening as the index date. We excluded the subjects who met the following exclusion criteria: (1) had missing data on smoking and alcohol intake questionnaire, measurements of fasting glucose, systolic blood pressure, diastolic blood pressure, and BMI; (2) diagnosed with cancer before index date; (3) answered “Yes” to cancer in disease history questionnaire; (4) had a follow-up duration of less than 1 year; and (5) answered “Yes” to HTN or DM history in disease history questionnaire- or was diagnosed with HTN [the International Classification of Diseases, 10th revision (ICD-10): ‘I10–15’] or DM (ICD-10: ‘E10–14’) (Fig. 1). Finally, the remaining 371,762 subjects were analyzed in this study (men, n = 205,876; women, n = 165,886).

### Definition

The study subjects were divided into normal, pre-hypertension, and undiagnosed hypertension groups according to BP. They were also classified as normal, IFG, and DM according to fasting serum glucose levels. For individuals without a history of hypertension, pre-hypertension was defined as a systolic blood pressure (SBP) between 120–139 mmHg or diastolic blood pressure (DBP) between 80–89 mmHg^7,18^. Undiagnosed hypertension was defined as subjects who were not aware of their hypertension nor taking antihypertensive medications, and those who presented with an SBP ≥ 140 mmHg or DBP ≥ 90 mmHg^19^. IFG was defined as a fasting plasma glucose level between 100 and 125 mg/dL by the 2003 American Diabetes Association^6^. Undiagnosed DM was defined as those who reported to not have been diagnosed with DM or prescribed DM medications by a physician, and who had a fasting plasma glucose of ≥ 126 mg/dL^20^. BMI was classified as three grades: underweight (<20 kg/m^2^), normal (20–24.9 kg/m^2^), and overweight (≥25.0 kg/m^2^)^21–23^. To assess other chronic medical conditions, Charlson comorbidity index was calculated using the claimed data for healthcare utilization during baseline^24^. Income level was categorized into medical aid beneficiaries, deciles for insured employees, and deciles for insured self-employed. Regarding the incident case of cancer, the first hospital admission with a cancer diagnostic code (ICD-10: C00-C96) was defined as the date of cancer occurrence during the follow-up period^25^. Based on the national cancer incidence statistics of South Korea^26^, a ranking of the specific-cancer type (1^st^–10^th^) in men and women was considered. Additionally, esophageal cancer was included in the analyses for men. The specific-cancer types were defined as follows: esophagus (C15), stomach (C16), colorectal (C18-C20), liver (C22), gallbladder (C23, C24), pancreas (C25), lung (C33, C34), prostate (C61), kidney (C64), bladder (C67), breast (C50), cervix uteri (C53), ovary (C56), and thyroid (C73) (Supplement Table 1).

### Statistical analysis

The follow-up period was calculated from the date of the health screening to the date of the diagnosis or death, or December 31, 2013. All analyzes were performed by a gender-stratification. The incidence rate of cancer was estimated as 100,000 person-years. The effect of pre-hypertension, an IFG, and the BMI on cancer risk was presented as a hazard ratio (HR) using a cox-proportional hazards model. A trend test was performed on the effect of BP and fasting glucose level on cancer risk. As covariates, the age at baseline, income level, smoking status, frequency of an alcohol intake, and Charlson comorbidity Index were considered. An assumption of the cox-proportional hazards model was evaluated through a log-log plot. The combination effect of pre-hypertension, an IFG, undiagnosed hypertension, and undiagnosed DM in men and women was presented focusing on five common cancer types (men: lung, prostate, colorectal, stomach, and liver, women: breast, colorectal, lung, cervix uteri, and stomach), with reference to the world cancer statistics^27^. Plus, we conducted subgroup analyses according to the BMI (<20 kg/m^2^, 20–24.9 kg/m^2^, and ≥25.0 kg/m^2^) in both men and women and also restricted the sample to never-smokers or non-alcohol drinkers (men, n = 41,015, women, n = 131,293). All statistical analyses were conducted using SAS ver. 9.4 software (SAS Institute, Cary, NC, USA). A two-sided *p*-value < 0.05 was considered significant.

### Ethics statement

This study protocol was approved by the institutional review board (IRB) of the Ewha Womans University Mokdong Hospital (IRB number: 2017–11–016). Informed consent was waived by the IRB because of the study’s retrospective nature. All methods of this study were performed in accordance with the relevant guidelines and regulations. Furthermore, this study was performed in compliance with the principles set forth in the Declaration of Helsinki.

## Results

### Baseline characteristics

The total of 371,762 subjects with complete medical data and who had answered questionnaires, consisted of 205,876 men (55.4%) and 165,886 women (44.6%). Of them, 35,605 (9.58%) developed cancer regardless of the site during a mean follow-up duration of 10.06 ± 1.86 years. The Table 1 shows the baseline characteristics of the study subjects.

**Table 1:** Baseline characteristics of the study subjects.

		Total	Men	Women
		(n = 371,762)	(n = 205,876)	(n = 165,886)
AGE, mean (S.D)	Years	51.22 (9.01)	50.72 (8.83)	51.84 (9.19)
Smoking	Never	242,190 (65.15)	82,457 (40.05)	159,733 (96.29)
	Previous	33,013 (8.88)	31,477 (15.29)	1,536 (0.93)
	Current	96,559 (25.97)	91,942 (44.66)	4,617 (2.78)
Alcohol intake	Never	203,249 (54.67)	68,921 (33.48)	134,328 (80.98)
	2,3 times/month	60,232 (16.2)	41,262 (20.04)	18,970 (11.44)
	1,2 times/week	64,755 (17.42)	55,404 (26.91)	9,351 (5.64)
	≥3 times/week	43,526 (11.71)	40,289 (19.57)	3,237 (1.95)
Percentile group of income level^†^	Medical, aid	594 (0.16)	228 (0.11)	366 (0.22)
	≤10th	31,953 (8.6)	13,126 (6.38)	18,827 (11.35)
	11,20th	24,828 (6.68)	11,094 (5.39)	13,734 (8.28)
	21,30th	24,955 (6.71)	11,112 (5.4)	13,843 (8.34)
	31,40th	26,273 (7.07)	13,187 (6.41)	13,086 (7.89)
	41,50th	28,260 (7.6)	15,335 (7.45)	12,925 (7.79)
	51,60th	30,826 (8.29)	17,944 (8.72)	12,882 (7.77)
	61,70th	35,679 (9.6)	21,384 (10.39)	14,295 (8.62)
	71,80th	41,988 (11.29)	25,097 (12.19)	16,891 (10.18)
	81,90th	59,578 (16.03)	35,574 (17.28)	24,004 (14.47)
	≥91th	66,828 (17.98)	41,795 (20.3)	25,033 (15.09)
CCI score, mean (S.D)		0.35 (0.65)	0.29 (0.61)	0.41 (0.7)
Systolic blood pressure	<120 mmHg	123,609 (33.25)	55,313 (26.87)	68,296 (41.17)
	120–139 mmHg	170,321 (45.81)	101,061 (49.09)	69,260 (41.75)
	≥140 mmHg	77,832 (20.94)	49,502 (24.04)	28,330 (17.08)
Diastolic blood pressure	<80 mmHg	160,039 (43.05)	72,668 (35.3)	87,371 (52.67)
	80–89 mmHg	129,480 (34.83)	77,684 (37.73)	51,796 (31.22)
	≥ 90 mmHg	82,243 (22.12)	55,524 (26.97)	26,719 (16.11)
Normal blood pressure		107,479 (28.91)	46,007 (22.35)	61,472 (37.06)
Prehypertension		158,511 (42.64)	91,209 (44.30)	67,302 (40.57)
Hypertension		105,772 (28.45)	68,660 (33.35)	37,112 (22.37)
Fasting serum glucose	<100 mg/dL	270,990 (72.89)	141,839 (68.9)	129,151 (77.86)
	100–125 mg/dL	83,990 (22.59)	52,154 (25.33)	31,836 (19.19)
	≥126 mg/dL	16,782 (4.51)	11,883 (5.77)	4,899 (2.95)
Body mass index	<20 kg/m^2^	220,799 (59.39)	119,846 (58.21)	100,953 (60.86)
	20–24.9 kg/m^2^	32,604 (8.77)	17,241 (8.37)	15,363 (9.26)
	≥25.0 kg/m^2^	118,359 (31.84)	68,789 (33.41)	49,570 (29.88)
All cancer incidence rate (per 100,000 person-years)		952.44	1063.58	815.15

Follow up period: 10.06 ± 1.86 years.

S.D, standard deviation; CCI, Charlson comorbidity Index; Normal blood pressure, SBP < 120 mmHg and DBP < 80 mmHg; prehypertension, 120 ≤ SBP < 140 mmHg or 80 < DBP < 90 mmHg; hypertension, SBP ≥ 140 mmHg or DBP ≥ 90 mmHg; ^†^Income level; The NHIS, health examination cohort provides income level data as medical aid beneficiaries, deciles for insured employees, and deciles for insured self, employed.

### Cancer risk according to the blood pressure, fasting serum glucose, and BMI

This study assessed the HR of various cancers according to factors including the BP, fasting serum glucose, and BMI in men and women over the follow-up duration (Table 2). In men, the risk of all cancers was significantly increased in the order of normal, pre-hypertension, and undiagnosed hypertension (pre-hypertension, HR = 1.01, 95% Confidence Interval [CI], 0.97–1.04; undiagnosed hypertension, HR = 1.06, 95%CI, 1.02–1.10; *P* for trend = 0.001). However, among the specific cancers, only the risk of colorectal cancer was shown to be significantly increased in the undiagnosed hypertension group (HR = 1.12, 95% CI, 1.02–1.23). In terms of the fasting serum glucose, the risk of all cancers had a significantly increased tendency with normal, IFG, and undiagnosed DM (IFG, HR = 1.05, 95% CI, 1.02–1.08; undiagnosed DM, HR = 1.24, 95% CI, 1.18–1.30; *P* for trend <0.001) in men. In terms of the BMI, the risk of all cancers was significantly higher in the groups with a BMI of <20 kg/m^2^ (HR = 1.14, 95% CI, 1.09–1.19) and ≥25.0 kg/m^2^ (HR = 1.03, 95% CI, 1–1.07) as compared to the 20–24.9 kg/m^2^ BMI group.

**Table 2:** Hazard ratio for specific cancers according to the blood pressure, fasting serum glucose, and body mass index level in Koreans, 2003–2013.

	Men				Women			
	Ref	HR (95% CI)	HR (95% CI)	*p* for trend	Ref	HR (95% CI)	HR (95% CI)	*p* for trend
**Blood pressure Pre-hypertension Undiagnosed hypertension Pre-hypertension Undiagnosed hypertension**								
**All cancer**	**1.00**	**1.01 (0.97,1.04)**	1.06 (1.02,1.10)^*^	**0.001**	1.00	1.04 (1.00,1.08)^*^	1.00 (0.96,1.05)	0.78
Stomach	1.00	0.99 (0.91,1.07)	1.03 (0.95,1.12)	0.35	1.00	1.02 (0.91,1.15)	0.97 (0.85,1.11)	0.72
**Colorectal**	**1.00**	**0.99 (0.90,1.09)**	1.12 (1.02,1.23)^*^	**<0.001**	1.00	0.97 (0.87,1.09)	0.98 (0.86,1.11)	0.70
Liver	1.00	1.00 (0.91,1.10)	1.08 (0.97,1.19)	0.11	1.00	1.09 (0.94,1.26)	1.16 (0.99,1.36)	0.07
Gall bladder	1.00	1.04 (0.82,1.33)	1.15 (0.90,1.47)	0.22	1.00	1.06 (0.82,1.36)	1.04 (0.79,1.37)	0.79
Pancreas	1.00	1.2 (0.97,1.49)	1.25 (0.99,1.56)	0.07	1.00	0.97 (0.76,1.24)	1.04 (0.80,1.36)	0.76
Lung	1.00	0.98 (0.89,1.08)	0.97 (0.88,1.07)	0.50	1.00	1.01 (0.88,1.16)	0.97 (0.84,1.13)	0.73
Thyroid	1.00	1.07 (0.89,1.29)	1.09 (0.89,1.33)	0.42	1.00	1.12 (1.03,1.21)^*^	1.01 (0.91,1.12)	0.37
Prostate	1.00	1.08 (0.97,1.20)	1.06 (0.95,1.19)	0.38				
Kidney	1.00	1.10 (0.84,1.44)	1.14 (0.86,1.52)	0.39				
Bladder	1.00	1.09 (0.88,1.37)	1.15 (0.91,1.44)	0.24				
Esophagus	1.00	0.92 (0.69,1.24)	1.04 (0.78,1.40)	0.63				
Breast					1.00	1.08 (0.97,1.2)	1.04 (0.91,1.19)	0.41
Cervix uteri					1.00	1.1 (0.88,1.38)	0.92 (0.70,1.21)	0.67
Ovary					1.00	0.98 (0.77,1.25)	1.07 (0.80,1.43)	0.71
**Fasting glucose**		**IFG**	**Undiagnosed DM**			**IFG**	**Undiagnosed DM**	
**All cancer**	**1.00**	1.05 (1.02,1.08)^*^	1.24 (1.18,1.30)^*^	**<0.001**	1.00	1.01 (0.97,1.05)	1.07 (0.97,1.17)	0.27
**Stomach**	**1.00**	**1.06 (0.99,1.14)**	1.21 (1.08,1.36)^*^	**0.001**	1.00	0.96 (0.84,1.08)	1.27 (1.01,1.61)^*^	0.44
**Colorectal**	**1.00**	**1.04 (0.96,1.12)**	1.22 (1.06,1.39)^*^	**0.01**	1.00	1.09 (0.97,1.23)	0.95 (0.73,1.23)	0.39
**Liver**	**1.00**	1.14 (1.05,1.24)^*^	1.74 (1.54,1.97)^*^	**<0.001**	1.00	1.01 (0.87,1.17)	1.17 (0.86,1.58)	0.49
**Gall bladder**	**1.00**	**1.12 (0.91,1.36)**	1.39 (1.01,1.92)^*^	**0.04**	1.00	1.1 (0.86,1.40)	1.27 (0.79,2.03)	0.25
Pancreas	1.00	1.03 (0.87,1.23)	1.32 (0.99,1.75)	0.13	1.00	0.99 (0.77,1.27)	1.46 (0.94,2.27)	0.31
Lung	1.00	1.02 (0.94,1.11)	1.13 (0.98,1.29)	0.12	1.00	0.97 (0.84,1.12)	0.84 (0.61,1.15)	0.32
Thyroid	1.00	1.03 (0.87,1.22)	0.64 (0.42,0.97)	0.23	1.00	1.05 (0.96,1.16)	0.98 (0.77,1.25)	0.45
Prostate	1.00	0.95 (0.86,1.04)	1.12 (0.96,1.31)	0.76				
Kidney	1.00	1.03 (0.81,1.30)	1.01 (0.65,1.57)	0.87				
Bladder	1.00	1.14 (0.95,1.36)	0.98 (0.69,1.39)	0.44				
Esophagus	1.00	1.04 (0.82,1.33)	1.14 (0.76,1.70)	0.51				
Breast					1.00	0.94 (0.83,1.07)	0.97 (0.71,1.32)	0.40
Cervix uteri					1.00	1.03 (0.80,1.32)	1.19 (0.71,2.00)	0.58
Ovary					1.00	0.97 (0.74,1.27)	0.81 (0.40,1.65)	0.60
**BMI, kg/m**^**2**^	**20–24.9**	**<20**	**≥25.0**		**20–24.9**	**<20**	**≥25.0**	
**All cancer**	**1.00**	1.14 (1.09,1.19)^*^	1.03 (1.00,1.07)^*^		1.00	1.01 (0.96,1.08)	1.03 (0.99,1.07)	
Stomach	1.00	1.10 (0.99,1.21)	1.01 (0.95,1.08)		1.00	1.1 (0.93,1.29)	1.01 (0.90,1.13)	
**Colorectal**	1.00	0.97 (0.86,1.09)	1.17 (1.09,1.26)^*^		1.00	0.83 (0.69,0.99)	1.05 (0.95,1.17)	
**Liver**	1.00	1.15 (1.02,1.29)^*^	1.02 (0.94,1.10)		1.00	0.89 (0.71,1.11)	1.06 (0.93,1.21)	
Gall bladder	1.00	0.90 (0.67,1.20)	1.16 (0.95,1.41)		1.00	1.37 (1.01,1.87)^*^	1.11 (0.88,1.39)	
Pancreas	1.00	1.15 (0.90,1.47)	1.07 (0.90,1.27)		1.00	1.29 (0.95,1.77)	1.06 (0.85,1.32)	
**Lung**	1.00	1.42 (1.29,1.56)^*^	0.81 (0.74,0.88)		1.00	1.09 (0.91,1.31)	0.86 (0.75,0.98)	
Thyroid	1.00	0.59 (0.40,0.87)	1.33 (1.15,1.54)		1.00	0.79 (0.68,0.9)	1.06 (0.98,1.16)	
Prostate	1.00	0.93 (0.81,1.06)	1.06 (0.97,1.15)					
Kidney	1.00	0.95 (0.64,1.42)	1.24 (0.99,1.54)					
**Bladder**	1.00	1.19 (0.93,1.54)	1.30 (1.09,1.56)^*^					
**Esophagus**	1.00	1.53 (1.15,2.04)^*^	0.73 (0.56,0.96)					
Breast					1.00	0.95 (0.8,1.13)	1.1 (0.99,1.22)	
Cervix uteri					1.00	1.53 (1.13,2.06)^*^	1.1 (0.88,1.37)	
Ovary					1.00	0.73 (0.47,1.13)	1.22 (0.97,1.53)	

HR, hazard ratio; CI, confidence interval; Ref, reference; IFG, impaired fasting glucose; DM, diabetic mellitus; SBP, systolic blood pressure; DBP, diastolic blood pressure;

Blood pressure, Ref = normal group (SBP < 120 mmHg and DBP < 80 mmHg), Pre-hypertension (120 ≤ SBP < 140 mmHg or 80 < DBP < 90 mmHg), and undiagnosed hypertension (SBP ≥ 140 mmHg or DBP ≥ 90 mmHg); Fasting glucose, Ref = Normal group (Fasting glucose < 100 mg/dL), IFG (Fasting glucose 100–125 mg/dL), and undiagnosed DM (Fasting glucose ≥126 mg/dL);

The hazard ratio was estimated from the subjects without a history of diabetes and hypertension.

The hazard ratio was obtained while controlling for the age, income level, smoking, alcohol intake, and Charlson comorbidity Index score; ^*^significant value, *P* < 0.05.

In women, there was no significant linear trend in cancer risk according to BP. The fasting serum glucose also did not show a linear trend of cancer in women. Only subjects with undiagnosed DM had a significantly increased risk of stomach cancer as compared to those with a normal fasting glucose (HR = 1.27, 95% CI, 1.01–1.61).

In terms of the BMI, the risk of gall bladder (HR = 1.37, 95% CI, 1.01–1.87) and cervix uteri (HR = 1.53, 95% CI, 1.13–2.06) cancer was significantly higher in the BMI < 20 kg/m^2^ group as compared to the 20–24.9 kg/m^2^ group.

### Cancer risk according to the combination of the blood pressure and fasting serum glucose

This study investigated cancer risk according to all combinations of BP and serum glucose level status **(**Table 3**)**. In men, subjects with undiagnosed hypertension and undiagnosed DM showed 1.29 HR (95% CI 1.20, 1.40) about all cancer risk compared with subjection with normal BP and glucose. Especially, all cancer risks showed an increased tendency with the addition in a dose-dependent manner of the fasting glucose. Among the subjects with pre-hypertension, all cancer risks serially increased when the subjects were accompanied by normal glucose, IFG, and undiagnosed DM (HR = 1.00, 95% CI, 0.96–1.05, *vs*. HR = 1.07, 95% CI, 1.02–1.13, *vs*. HR = 1.26, 95% CI, 1.16–1.37). Likewise, among the subjects with undiagnosed hypertension, all cancer risks also serially increased when the subjects were accompanied by normal glucose, IFG, and undiagnosed DM (HR = 1.07, 95% CI, 1.03–1.12, *vs*. HR = 1.07, 95% CI, 1.02–1.13, *vs*. HR = 1.29, 95% CI, 1.20–1.40). However, the addition of BP did not show an increased tendency for all cancer risks in a dose-dependent manner. When analyzed among subjects with an IFG, all cancer risks did not significantly increase when the subjects were accompanied by a normal BP, prehypertension, and undiagnosed hypertension (HR = 1.09, 95% CI, 1.02–1.17, *vs*. HR = 1.07, 95% CI, 1.02–1.13, *vs*. HR = 1.07, 95% CI, 1.02–1.13). This pattern was also observed in the undiagnosed hypertension group.

**Table 3:** Cancer risk according to the combination of the blood pressure and fasting serum glucose.

Risk factors		Men						Women					
Blood pressure	Fasting glucose	All cancer	Lung	Prostate	Colorectal	Stomach	Liver	All cancer	Breast	Colorectal	Lung	Cervix uteri	Stomach
**−**	**−**	1.00	1.00	1.00	1.00	1.00	1.00	1.00	1.00	1.00	1.00	1.00	1.00
**−**	**+**	1.09^*^ **(1.02,1.17)**	1.1 (0.91,1.32)	1.14 (0.92,1.41)	1 (0.83,1.21)	1.2 (1.03,1.4)	1.06 (0.88,1.28)	1.02 (0.95,1.1)	0.78 (0.61,0.99)	1.14 (0.92,1.4)	0.96 (0.73,1.26)	1.23 (0.81,1.88)	0.98 (0.78,1.24)
**−**	**++**	1.2^*^ **(1.04,1.39)**	1.2 (0.84,1.72)	0.78 (0.45,1.32)	1.31 (0.92,1.87)	1.45^*^ **(1.08,1.95)**	1.22 (0.83,1.79)	0.96 (0.78,1.19)	1.01 (0.54,1.89)	0.74 (0.38,1.43)	0.64 (0.29,1.43)	0.39 (0.05,2.78)	1.05 (0.59,1.86)
**+**	**−**	1 (0.96,1.05)	0.99 (0.89,1.11)	1.11 (0.98,1.26)	0.98 (0.87,1.09)	1.03 (0.94,1.13)	0.94 (0.84,1.05)	1.05 (1.00,1.09)	1.05 (0.94,1.18)	0.99 (0.87,1.12)	0.98 (0.84,1.15)	1.15 (0.89,1.47)	1.02 (0.89,1.16)
**+**	**+**	1.07^*^ **(1.02,1.13)**	0.99 (0.86,1.13)	1.01 (0.86,1.19)	1.04 (0.91,1.19)	1.09 (0.97,1.23)	1.11 (0.97,1.28)	1.02 (0.95,1.09)	1.05 (0.87,1.26)	0.99 (0.82,1.2)	1.03 (0.82,1.28)	1.12 (0.77,1.64)	1.01 (0.83,1.22)
**+**	**++**	1.26^*^ **(1.16,1.37)**	1.32^*^ **(1.07,1.63)**	1.42^*^ **(1.1,1.81)**	1.21 (0.97,1.52)	1.02 (0.83,1.27)	1.67^*^ **(1.36,2.05)**	1.08 (0.93,1.25)	0.71 (0.4,1.26)	1.03 (0.69,1.54)	0.98 (0.61,1.58)	0.83 (0.30,2.24)	1.17 (0.79,1.74)
**++**	**−**	1.07^*^ **(1.03,1.12)**	0.99 (0.88,1.12)	1.11 (0.97,1.27)	1.12 (1.00,1.25)	1.07 (0.97,1.18)	1 (0.88,1.12)	0.98 (0.93,1.04)	0.97 (0.83,1.14)	0.95 (0.82,1.11)	0.99 (0.83,1.18)	0.9 (0.65,1.25)	0.97 (0.83,1.13)
**++**	**+**	1.07^*^ **(1.02,1.13)**	1.02 (0.88,1.17)	1.01 (0.85,1.19)	1.12 (0.98,1.29)	1.07 (0.95,1.21)	1.12 (0.97,1.29)	1.03 (0.96,1.11)	1.04 (0.82,1.33)	1.13 (0.92,1.38)	0.89 (0.69,1.15)	0.81 (0.49,1.35)	0.87 (0.70,1.09)
**++**	**++**	1.29^*^ **(1.20,1.40)**	0.95 (0.76,1.18)	1.18 (0.93,1.5)	1.27^*^ **(1.04,1.56)**	1.38^*^ **(1.17,1.64)**	1.85^*^ **(1.54,2.22)**	1.15 (0.99,1.33)	1.29 (0.81,2.07)	0.94 (0.63,1.41)	0.79 (0.48,1.29)	2.09^*^ **(1.09,4.02)**	1.45^*^ **(1.03,2.05)**

‘–’; normal group, ‘+’; pre-hypertension/ impaired fasting glucose, ‘++’; undiagnosed hypertension/undiagnosed diabetic mellitus

The results are presented as the hazard ratio with a 95% confidence interval. The hazard ratio was estimated from the subjects without a history of diabetes and hypertension.

Blood pressure - normal group (SBP < 120 mmHg and DBP < 80 mmHg), pre-hypertension (120≤SBP < 140 mmHg or 80 < DBP < 90 mmHg), and undiagnosed hypertension (SBP ≥ 140 mmHg or DBP ≥ 90 mmHg); Fasting glucose - Normal group (Fasting glucose <100 mg/dL), impaired fasting glucose (Fasting glucose 100–125 mg/dL), and undiagnosed diabetic mellitus (Fasting glucose ≥126 mg/dL); ^*^significant value, *P* < 0.05.

However, in women, BP and fasting serum glucose level status did not show any effect on all cancer risks. Further, the combination of BP and fasting serum glucose level status also did not increase all cancer risks. Only the subjects with both undiagnosed hypertension and undiagnosed DM increased the risk of cervix uteri and stomach cancer as compared to the normal group (HR = 2.09, 95% CI, 1.09–4.02, HR = 1.45, 95% CI, 1.03–2.05).

### Cancer risk according to the blood pressure and fasting serum glucose stratified by the BMI

**Figure 2:**
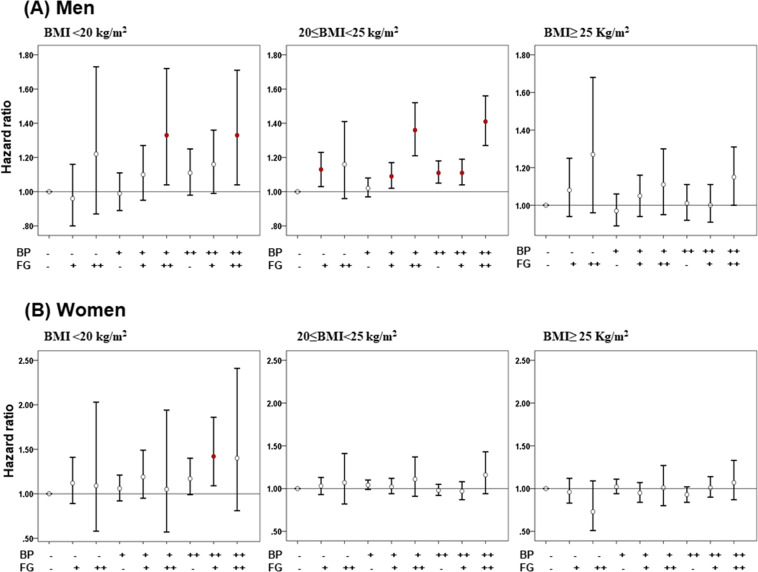
Cancer risk according to the blood pressure and fasting serum glucose levels stratified by the body mass index. (**A**) men, (**B**) women. Abbreviations: BMI, body mass index; BP, blood pressure; FG, fasting glucose; ‘−’; normal group, ‘+’; pre-hypertension/ impaired fasting glucose, ‘++’; undiagnosed hypertension/undiagnosed diabetic mellitus. Blood pressure, normal group (SBP < 120 mmHg and DBP < 80 mmHg), Pre-hypertension (120 ≤ SBP < 140 mmHg or 80 < DBP < 90 mmHg), and undiagnosed hypertension (SBP ≥ 140 mmHg or DBP ≥ 90 mmHg); Fasting glucose, normal group (Fasting glucose < 100 mg/dL), IFG (Fasting glucose 100–125 mg/dL), and undiagnosed DM (Fasting glucose ≥ 126 mg/dL).

To control the effect of BMI on cancer risk, we conducted a subgroup analysis in both men and women after the study subjects were divided into underweight (<20 kg/m^2^), normal (20–24.9 kg/m^2^), and overweight (≥25.0 kg/m^2^) subgroups (Fig. 2). In the men in the underweight group, the overall cancer risk significantly increased only in those with undiagnosed DM accompanied with pre-hypertension or undiagnosed hypertension. In the normal BMI group, all cancer risks demonstrated an increased tendency with the addition in a dose-dependent manner of the fasting glucose. This result was consistent with the aforementioned (Table 2). When analyzed only in those with pre-hypertension or undiagnosed hypertension, all cancer risks were serially increased when accompanied by IFG or undiagnosed DM (red dot in Fig. 2A). However, when analyzed only in those with an IFG or undiagnosed DM, the increased tendency of a cancer risk was not significant with the addition of pre-hypertension or undiagnosed hypertension (Fig. 2A). In contrast, in women, there was no significant difference in cancer risks according to BP and fasting serum glucose, except for undiagnosed hypertension and IFG in the underweight group (Fig. 2B).

### Cancer risk according to the blood pressure and fasting serum glucose excluding the effects of smoking and alcohol intake

**Figure 3:**
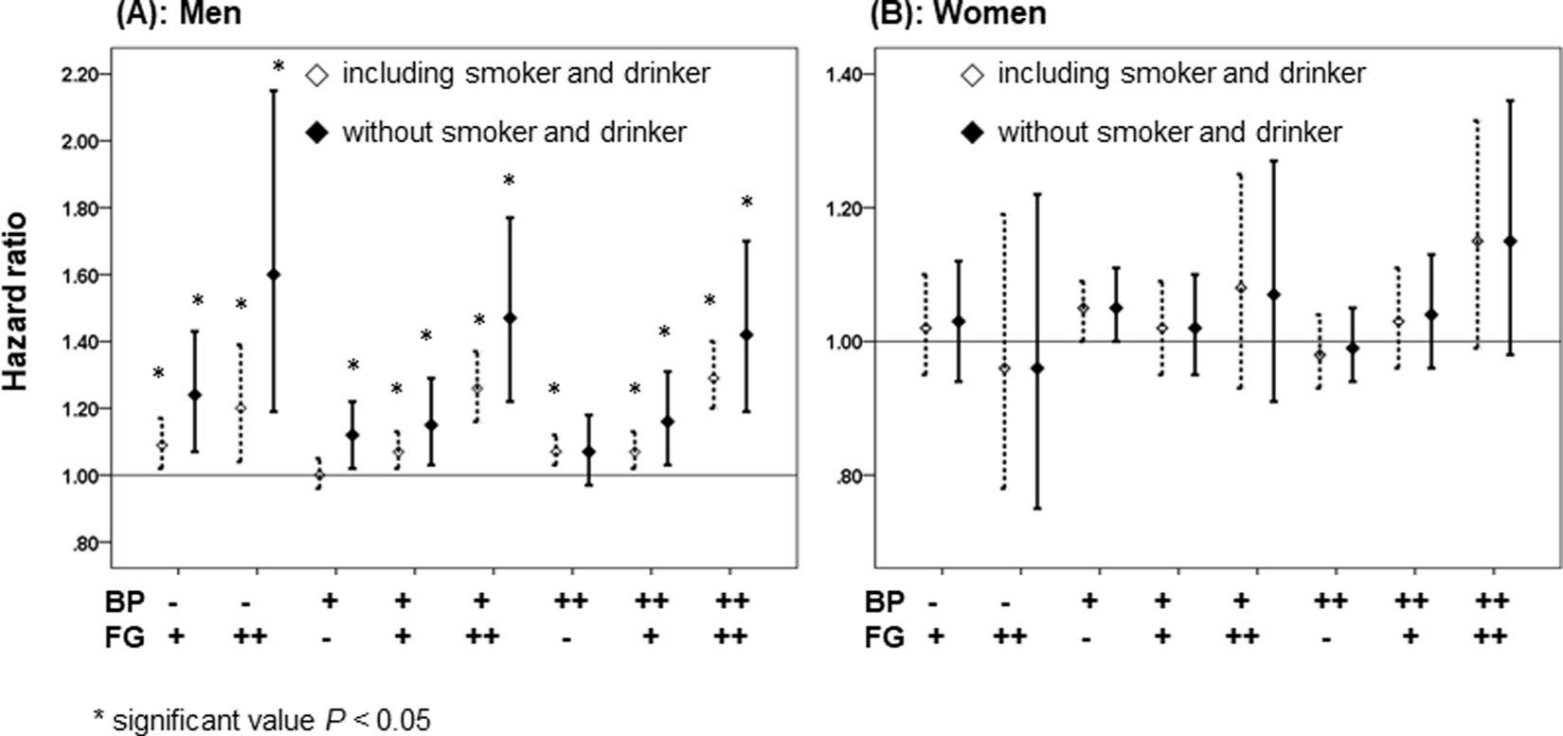
Comparison of cancer risk according to the blood pressure and fasting serum glucose between groups with smokers and drinkers and without. (**A**) men, (**B**) women. Abbreviations: BP, blood pressure; FG, fasting glucose; ‘−’; normal group, ‘+’; pre-hypertension/ impaired fasting glucose, ‘++’; undiagnosed hypertension/undiagnosed diabetic mellitus. Blood pressure, normal group (SBP < 120 mmHg and DBP < 80 mmHg), Pre-hypertension (120 ≤ SBP < 140 mmHg or 80 < DBP < 90 mmHg), and undiagnosed hypertension (SBP ≥ 140 mmHg or DBP ≥ 90 mmHg); Fasting glucose, normal group (Fasting glucose <100 mg/dL), IFG (Fasting glucose 100–125 mg/dL), and undiagnosed DM (Fasting glucose ≥126 mg/dL).

Because a difference in the results according to the gender may be influenced by smoking and drinking, we conducted an additional analysis of cancer risk according to a combination of BP and fasting serum glucose level status in only never smoker and non-alcohol drinker subjects. In the results of that analysis, among the men with pre-hypertension or undiagnosed hypertension, all cancer risks serially increased with the addition of IFG or undiagnosed DM in a dose-dependent manner (Supplement Table 2). However, no significant association was seen in women. In addition, when BP and fasting serum glucose level status was the same, the analysis results of men who were never-smokers and non-alcohol drinkers exhibited a higher HR than that of men who were smokers and drinkers (Fig. 3A). However, there was no significant difference in the analysis of women (Fig. 3B). Furthermore, we re-analyzed cancer risk according to BMI in only never smoker and non-alcohol drinker subjects. The results showed that in men with pre-hypertension or undiagnosed hypertension, all cancer risks serially increased with the addition of IFG or undiagnosed DM in a dose-dependent manner, especially normal BMI (20–24.9 kg/m^2^). However, no significant association was observed in women (Supplement Table 3). Hazard ratio for specific cancers according to body mass index level in subjects without a smoking and alcohol history are shown Supplement Table 4. In men, there was no significant risk in the groups with BMI < 20 kg/m^2^. However, in the BMI ≥ 25.0 kg/m^2^ group, cancer risk still increased. In women, all cancer risks did not change, while ovary cancer risk increased in the BMI ≥ 25.0 kg/m^2^ group.

## Discussion

In this study, we confirmed that an increased BP status (pre-hypertension, undiagnosed hypertension) or the fasting serum glucose level status (IFG, undiagnosed DM) were associated with cancer risk in men. When analyzing the combination effect of BP and fasting serum glucose level status, all cancer risks were serially increased with an increase in the fasting serum glucose level in a dose-dependent manner, but not with an increase in BP status. In addition, the combination of both pre-hypertension and an IFG also was associated with cancer risk in men, which was most prominent in the normal BMI group. However, there was no significant association between BP or fasting glucose level and cancer risk in women. Furthermore, when removing the effects of smoking and drinking, a combination of pre-hypertension and an IFG were more associated with cancer risk in men.

A previous large European cohort study (n = 577,799) reported the association between BP and cancer risk in men and no association in women, similar to our study of an Asian population^9^. Another large European study (n = 307,318) also reported the association among SBP, DBP, and cancer risk^28^. Our additional analysis also showed that cancer risk increased with 10-mmHg increments in both SBP and DBP in men, but not in women (SBP, HR = 1.02, 95% CI, 1.01–1.02, DBP, HR = 1.02, 95% CI, 1.0–1.1 Supplement Table 5). However, those studies did not distinguish between pre-hypertension and hypertension; and instead, cancer risk was investigated by quintiles and 10-mmHg increments of the mid-blood pressure. Therefore, our study focused more on the association of cancer risk with pre-hypertension compared to previous studies. In specific cancer type incidences, the colorectal cancer risk showed a similar association with hypertension as in the other studies^2,9,29^. The colorectal cancer risk was also associated with men with a BMI of ≥ 25.0 kg/m^2^ and elevated fasting serum glucose level, and those results suggest that colorectal cancer is associated with metabolic syndrome as supported by another study^2^. In contrast, many large cohort studies have reported that BP is associated with kidney cancer in men^9,30,31^, however, our study did not show any significant association between BP and kidney cancer. A study reported that antihypertensive medications were reported to be associated with kidney cancer^32^. Our study consisted of subjects who did not take antihypertensive medications at the time of enrollment, and this may have affected the results. Many studies reported that the fasting serum glucose level was associated with a cancer risk^3,10–12,33–36^, and our study showed similar results in men. However, our study did not show a significant association between the fasting serum glucose level and risk of all cancers in women. Although a previous study from a Korean cohort showed that the pancreatic and liver cancer risks were associated with the fasting serum glucose level in women, all cancer risks were not associated^11^. In contrast, a European large scale study not involving Asians showed a higher risk of all cancers in women than in men^36^. Our study showed that only men had a meaningful association between a cancer risk and the fasting serum glucose level, which may have been affected by the race, life style, and glucose definition of the study design.

This study showed that BP status and fasting glucose level status were associated with cancer risk in only men, but not in women. In these study subjects, men were more likely to smoke and consume alcohol than women, and this could have affected the results since smoking and alcohol intake are well-known risk factors of cancer^14–16^. However, even among never-smokers and non-alcohol drinkers, we found that an increased BP status and the fasting glucose level status were still associated with cancer risk in only men. This result showed that a gender difference may directly affect cancer risk with BP and fasting serum glucose level, while lifestyle choices such as smoking or alcohol intake would not.

In addition, one of the important points is that men with pre-hypertension and an IFG with a normal BMI, who did not need any hypertension or DM medication, could be at an increased risk of cancer (Fig. 2A). Because pre-hypertension and IFG are conditions that can be overcome by lifestyle modifications without medications, the association among pre-hypertension, an IFG, and cancer risk indicate the importance of controlling BP and fasting serum glucose in men.

The recent hypertension guidelines in the 2017 ACC/AHA defined hypertension as an SBP above 130 mmHg or DBP above 80 mmHg, and those values are lower than the previous JNC-8 and 2018 ESC/ESH guidelines^7,18,37^. Furthermore, the 2017 ACC/AHA guidelines recommend treatment for an SBP above 130 mmHg or DBP above 80 mmHg using antihypertensive medications if subjects have risk factors including DM^37^. Of course, the changes in the guidelines were based on the cardiovascular risk and mortality, but it could also be significant in terms of cancer risk, based on this study result that men with both pre-hypertension and an IFG had a higher cancer risk.

However, our study had its limitations. First, our study used a homogeneous Asian cohort, and therefore our findings may not be generalized. The reproducibility and applicability should be tested by future studies in other cohorts as well. Second, BP should be measured at least twice over at least two days to diagnose hypertension, but many subjects in this cohort study were measured on a single day. Therefore, some of the normal or pre-hypertension subjects could be included in the hypertension group, which may be accompanied by a misclassification bias. Third, at the time of enrollment, although subjects with undiagnosed hypertension or undiagnosed DM did not take DM or hypertension medications, we could not consider that some subjects took DM or hypertension medications during the study after enrollment. Finally, the occupation, living environment, lifestyle and physical activity could have influenced the incidence of a cancer incidence. Unfortunately, we could not analyze the effect of those factors.

## Conclusions

Both the BP level and an increased fasting serum glucose level themselves were significantly associated with all cancer risks in men, but not in women. Further, the combination of pre-hypertension and IFG increased the all cancer risks in men, especially in the normal BMI group, which persisted when removing the effects of smoking and alcohol drinking. Therefore, even normal BMI men without HTN and DM must pay attention to their BP and fasting serum glucose through lifestyle modifications if they have pre-hypertension and IFG. In addition, this difference in the cancer risk between men and women might be another unknown risk factor related to gender, and not due to differences in smoking and alcohol intake.

## Supplementary information

Supplementary Information.
