# Supplementary material for: Clinical impact of pre-hypertension on the risk of cancer in male and female subjects
Source: Sci Rep. 2020 Jun 19;10:9974. doi: 10.1038/s41598-020-66653-y (PMC7305195; doi:10.1038/s41598-020-66653-y)
Supplement: Supplementary file 1 — Supplementary Information. [file 41598_2020_66653_MOESM1_ESM.docx]

**Supplementary Information**

**Clinical impact of pre-hypertension on the risk of cancer in male and female subjects**

Su Hwan Lee^1^**^†^**, Hye Ah Lee^2,3^**^†^**, Sean S. Lee^4^, Seong-Eun Kim^5^, Ki-Nam Shim^5^, Hye-Kyung Jung^5^, Sung-Ae Jung^5^, Jung Hyun Chang^6^, Kihwan Kwon^7^, Wook Bum Pyun^7^, Boyoung Joung^8^, Chang Mo Moon^5,9^**^*^**^‡^, and Junbeom Park^7^**^*^**^‡^

**Supplementary Methods Section 1**

The total population in the Republic of Korea are obligated to enroll in the NHIS, a single-insurer system for medical care, and all participants’ data, including the insurance eligibility, medical treatment, and medical care institutions, are stored in the NHIS data base. The NHIS regularly conducts nationwide heath examinations including blood laboratory tests, chest x-ray examinations, physical examinations, and medical questionnaires for participants of ages 40 years and above, insured employees, householders of the insured self-employed, and blue-collar employees. That data are merged and compose the NHIS-Health Screening Cohort; it contains data of all participants followed up to December 31, 2013. Detailed information about the health screening data can be found in a previous study.^1^ Further, we designed this study with reference to the previous studies conducted by our researchers.^2^

**Reference**

1. Seong SC, Kim YY, Park SK, et al. Cohort profile: the National Health Insurance Service-National Health Screening Cohort (NHIS-HEALS) in Korea. *BMJ Open.* 2017;7(9):e016640.

2. Lee SS, Ae Kong K, Kim D, et al. Clinical implication of an impaired fasting glucose and prehypertension related to new onset atrial fibrillation in a healthy Asian population without underlying disease: a nationwide cohort study in Korea. *Eur Heart J.* 2017;38(34):2599-2607.

**Supplement Table 1. Cancer codes according to ICD 10 code**

|  | ICD-10 code |
| --- | --- |
| All cancer | C00-C96 |
| Stomach | C16 |
| Colorectal | C18 -C20 |
| Liver | C22 |
| Gall bladder | C23-C24 |
| Pancreas | C25 |
| Lung | C33-C34 |
| Thyroid | C73 |
| Prostate | C61 |
| Kidney | C64 |
| Bladder | C67 |
| Esophagus | C15 |
| Breast | C50 |
| Cervix uteri | C53 |
| Ovary | C56 |

**Supplement Table 2. Cancer risk according to a combination of risk factors in subjects without a smoking and alcohol history**

| Risk factors | | Men | | | | | |  | Women | | | | | |
| --- | --- | --- | --- | --- | --- | --- | --- | --- | --- | --- | --- | --- | --- | --- |
| Blood pressure | Fasting glucose | **All cancer** | Lung | Prostate | Colorectal | Stomach | Liver |  | **All**  **cancer** | Breast | Colorectal | Lung | Cervix uteri | Stomach |
| **-** | **-** | **1.00** | 1.00 | 1.00 | 1.00 | 1.00 | 1.00 |  | 1.00 | 1.00 | 1.00 | 1.00 | 1.00 | 1.00 |
| **-** | **+** | **1.24^*^ (1.07,1.43)** | 1.40 (0.96,2.04) | 1.06 (0.71,1.60) | 1.10 (0.73,1.67) | 1.36 (0.98,1.88) | 1.06 (0.69,1.60) |  | 1.03 (0.94,1.12) | 0.79 (0.60,1.03) | 1.18 (0.93,1.49) | 0.89 (0.65,1.23) | 1.09 (0.65,1.81) | 1.01 (0.78,1.30) |
| **-** | **++** | **1.60^*^ (1.19,2.15)** | 1.21 (0.49,2.99) | 1.17 (0.48,2.85) | **2.02^*^ (1.00,4.10)** | 1.23 (0.57,2.66) | **3.25^*^ (1.81,5.82)** |  | 0.96 (0.75,1.22) | 1.24 (0.66,2.32) | 0.62 (0.28,1.40) | 0.43 (0.14,1.34) | 0.51 (0.07,3.62) | 1.07  (0.57,2.01) |
| **+** | **-** | **1.12^*^ (1.02,1.22)** | 0.98 (0.76,1.25) | 1.15 (0.91,1.45) | 1.16 (0.91,1.47) | 1.08 (0.88,1.33) | 1.10 (0.86,1.40) |  | 1.05 (1.00,1.11) | 1.07 (0.93,1.22) | 1.01 (0.88,1.17) | 0.95 (0.80,1.13) | 1.15 (0.86,1.53) | 0.98 (0.84,1.13) |
| **+** | **+** | **1.15^*^ (1.03,1.29)** | 1.14 (0.84,1.56) | 0.90 (0.65,1.24) | 1.10 (0.80,1.50) | 1.18 (0.91,1.52) | 1.26 (0.93,1.71) |  | 1.02 (0.95,1.10) | 0.99 (0.80,1.24) | 1.01 (0.82,1.24) | 1.02 (0.80,1.31) | 1.09 (0.71,1.69) | 1.03 (0.83,1.27) |
| **+** | **++** | **1.47^*^ (1.22,1.77)** | 1.32 (0.79,2.20) | **2.24^*^ (1.49,3.38)** | 1.43 (0.85,2.39) | 1.41 (0.91,2.17) | 1.14 (0.64,2.02) |  | 1.07 (0.91,1.27) | 0.74 (0.40,1.38) | 1.08 (0.69,1.67) | 0.81 (0.45,1.45) | 1.07 (0.39,2.92) | 1.21 (0.79,1.85) |
| **++** | **-** | 1.07 (0.97,1.18) | 0.94 (0.72,1.23) | 1.01 (0.78,1.30) | 1.12 (0.86,1.45) | 1.10 (0.89,1.38) | 0.97 (0.74,1.27) |  | 0.99 (0.94,1.05) | 0.91 (0.76,1.09) | 0.95 (0.80,1.12) | 0.96 (0.79,1.16) | 0.91 (0.63,1.31) | 0.97 (0.82,1.15) |
| **++** | **+** | **1.16^*^ (1.03,1.31)** | 0.80 (0.56,1.14) | 0.99 (0.72,1.37) | 1.28 (0.94,1.75) | 1.23 (0.94,1.60) | 1.21 (0.87,1.67) |  | 1.04 (0.96,1.13) | 1.04 (0.79,1.36) | 1.09 (0.87,1.37) | 0.82 (0.61,1.09) | 0.88 (0.50,1.55) | 0.90 (0.71,1.15) |
| **++** | **++** | **1.42^*^ (1.19,1.70)** | 0.89 (0.50,1.57) | 0.99 (0.58,1.69) | 1.55 (0.97,2.46) | 1.15 (0.75,1.78) | **2.45^*^ (1.64,3.66)** |  | 1.15 (0.98,1.36) | 1.34 (0.80,2.25) | 0.89  (0.56,1.41) | 0.82 (0.48,1.42) | 1.93 (0.89,4.19) | **1.47^*^ (1.01,2.14)** |

‘–’; normal group, ‘+’; pre-hypertension/ impaired fasting glucose, ‘++’; undiagnosed hypertension/undiagnosed diabetic mellitus

The results are presented as the hazard ratio with a 95% confidence interval. The hazard ratio was estimated from the subjects without a history of diabetes and hypertension.

Blood pressure - normal group (SBP<120 mmHg and DBP <80 mmHg), pre-hypertension (120<SBP<140 mmHg or 80<DBP<90 mmHg), and undiagnosed hypertension (SBP≥140 mmHg or DBP≥90 mmHg); Fasting glucose - Normal group (Fasting glucose <100 mg/dL), impaired fasting glucose (Fasting glucose 100-125 mg/dL), and undiagnosed diabetic mellitus (Fasting glucose ≥ 126 mg/dL); ^*^significant value, *P* < 0.05

**Supplement Table 3. Blood pressure and Fasting glucose combination and cancer risk according to BMI in subjects without a smoking and alcohol history**

| Risk factors | | | Men | Women |
| --- | --- | --- | --- | --- |
| **BMI** | Blood pressure | Fasting glucose | HR (95% CI) | HR (95% CI) |
| BMI<20 | **-** | **-** | ref | ref |
| BMI<20 | **-** | **+** | 1.27 (0.86,1.87) | 1.1 (0.84,1.44) |
| BMI<20 | **-** | **++** | 0.67 (0.27,1.66) | 0.79 (0.32,1.93) |
| BMI<20 | **+** | **-** | 1.05 (0.84,1.32) | 1.08 (0.92,1.26) |
| BMI<20 | **+** | **+** | 1.05 (0.74,1.49) | 1.23 (0.96,1.59) |
| BMI<20 | **+** | **++** | **2.06 (1.12,3.77)** | 1.39 (0.75,2.59) |
| BMI<20 | **++** | **-** | 0.93 (0.71,1.23) | 1.18 (0.97,1.44) |
| BMI<20 | **++** | **+** | 1.27 (0.88,1.82) | **1.51 (1.12,2.03)** |
| BMI<20 | **++** | **++** | 0.96 (0.42,2.18) | **1.82 (1.04,3.18)** |
| 20≤BMI<25 | **-** | **-** | ref | ref |
| 20≤BMI<25 | **-** | **+** | **1.24 (1.03,1.49)** | 1.04 (0.93,1.16) |
| 20≤BMI<25 | **-** | **++** | **1.93 (1.33,2.8)** | 1.14 (0.85,1.53) |
| 20≤BMI<25 | **+** | **-** | **1.13 (1,1.27)** | 1.04 (0.98,1.11) |
| 20≤BMI<25 | **+** | **+** | **1.19 (1.03,1.39)** | 1.03 (0.93,1.13) |
| 20≤BMI<25 | **+** | **++** | **1.74 (1.38,2.21)** | 1.09 (0.86,1.37) |
| 20≤BMI<25 | **++** | **-** | **1.15 (1.02,1.31)** | 0.98 (0.9,1.05) |
| 20≤BMI<25 | **++** | **+** | **1.17 (1,1.37)** | 0.96 (0.85,1.08) |
| 20≤BMI<25 | **++** | **++** | **1.42 (1.11,1.84)** | 1.16 (0.91,1.47) |
| 20≤BMI<25 | **-** | **-** | ref | ref |
| BMI≥25 | **-** | **+** | 1.22 (0.91,1.64) | 0.97 (0.82,1.14) |
| BMI≥25 | **-** | **++** | 1.6 (0.91,2.81) | 0.71 (0.45,1.12) |
| BMI≥25 | **+** | **-** | 1.1 (0.92,1.33) | 1.04 (0.95,1.15) |
| BMI≥25 | **+** | **+** | 1.09 (0.87,1.35) | 0.95 (0.83,1.09) |
| BMI≥25 | **+** | **++** | 1.01 (0.71,1.44) | 0.98 (0.75,1.28) |
| BMI≥25 | **++** | **-** | 0.96 (0.8,1.17) | 0.95 (0.86,1.06) |
| BMI≥25 | **++** | **+** | 1.1 (0.89,1.37) | 1.04 (0.91,1.19) |
| BMI≥25 | **++** | **++** | **1.42 (1.07,1.89)** | 1.05 (0.83,1.33) |

The results are presented as the hazard ratio (HR) with a 95% confidence interval (CI).

‘–’; normal group, ‘+’; pre-hypertension/ impaired fasting glucose, ‘++’; undiagnosed hypertension/undiagnosed diabetic mellitus. Reference (Ref): Blood pressure, normal group (SBP<120 mmHg and DBP <80 mmHg), pre-hypertension (120<SBP<140 mmHg or 80<DBP<90 mmHg), and undiagnosed hypertension (SBP≥140 mmHg or DBP≥90 mmHg); Fasting glucose, normal group (Fasting glucose <100 mg/dL), impaired fasting glucose (Fasting glucose 100-125 mg/dL), and undiagnosed diabetic mellitus (Fasting glucose ≥ 126 mg/dL)

**Supplement Table 4. Hazard ratio for specific cancers according to body mass index level in subjects without a smoking and alcohol history**

HR, hazard ratio; CI, confidence interval; Ref, reference

The hazard ratio was estimated from the subjects without a history of diabetes and hypertension.

The hazard ratio was obtained while controlling for the age, income level, smoking, alcohol intake, and Charlson comorbidity Index score; ^*^ significant value, *P* < 0.05

|  | **Men** | | | | **Women** | | |
| --- | --- | --- | --- | --- | --- | --- | --- |
|  | **Ref** | | **HR (95% CI)** | **HR (95% CI)** | **Ref** | **HR (95% CI)** | **HR (95% CI)** |
| **Body Mass Index** | | **20-24.9 kg/m^2^** | **< 20 kg/m^2^** | **≥ 25.0 kg/m^2^** | **20-24.9 kg/m^2^** | **< 20 kg/m^2^** | **≥ 25.0 kg/m^2^** |
| All cancer | 1.00 | | 1.05 (0.95,1.15) | **1.07**^*^ **(1.01,1.14)** | 1.00 | 1.00 (0.93,1.06) | 1.03 (0.99,1.08) |
| Stomach | 1.00 | | 0.90 (0.72,1.13) | 1.00 (0.87,1.16) | 1.00 | 1.09 (0.90,1.31) | 1.00 (0.89,1.13) |
| Colorectal | 1.00 | | 1.04 (0.80,1.35) | **1.20**^*^ **(1.02,1.42)** | 1.00 | 0.82 (0.67,1.01) | 1.08 (0.97,1.21) |
| Liver | 1.00 | | 1.03 (0.79,1.36) | 1.11 (0.93,1.32) | 1.00 | 0.85 (0.66,1.09) | 1.04 (0.90,1.20) |
| Gall bladder | 1.00 | | 0.80 (0.40,1.58) | 1.40 (0.94,2.07) | 1.00 | 1.24 (0.86,1.79) | 1.19 (0.93,1.52) |
| Pancreas | 1.00 | | 1.18 (0.70,2.02) | 1.19 (0.83,1.70) | 1.00 | 1.31 (0.92,1.86) | 1.14 (0.90,1.45) |
| Lung | 1.00 | | **1.40**^*^ **(1.11,1.76)** | 0.82 (0.67,0.99) | 1.00 | 1.16 (0.94,1.43) | 0.88 (0.76,1.02) |
| Thyroid | 1.00 | | 0.51 (0.21,1.26) | **1.79**^*^ **(1.32,2.44)** | 1.00 | 0.74 (0.64,0.87) | 1.07 (0.97,1.17) |
| Prostate | 1.00 | | 0.99 (0.76,1.28) | 1.17 (0.99,1.38) |  |  |  |
| Kidney | 1.00 | | 0.81 (0.37,1.81) | 1.26 (0.82,1.94) |  |  |  |
| Bladder | 1.00 | | 1.07 (0.63,1.83) | 1.04 (0.70,1.54) |  |  |  |
| Esophagus | 1.00 | | 1.19 (0.47,3.04) | 1.13 (0.57,2.22) |  |  |  |
| Breast |  | |  |  | 1.00 | 0.90 (0.74,1.09) | 1.09 (0.97,1.23) |
| Cervix uteri |  | |  |  | 1.00 | **1.47**^*^ **(1.04,2.07)** | 1.02 (0.80,1.32) |
| Ovary |  | |  |  | 1.00 | 0.62 (0.36,1.07) | **1.37**^*^ **(1.06,1.77)** |

**Supplement Table 5. Hazard ratio for specific cancers according to systolic and diastolic blood pressure.**

|  | Men | | | | Women | | | |
| --- | --- | --- | --- | --- | --- | --- | --- | --- |
|  | SBP(per 10mmHg) | | DBP(per 10mmHg) | | SBP(per 10mmHg) | | DBP(per 10mmHg) | |
|  | HR  (95% CI) | *p* | HR  (95% CI) | *p* | HR  (95% CI) | *p* | HR  (95% CI) | *p* |
| All cancer | **1.02**^*^ **(1.01,1.02)** | **<0.001** | **1.02**^*^  **(1,1.03)** | **0.01** | 1.00 (0.99,1.01) | 0.93 | 1.00 (0.99,1.02) | 0.86 |
| Stomach | 1.01 (0.99,1.03) | 0.36 | 1.01 (0.99,1.04) | 0.38 | 0.97 (0.95,1.00) | 0.06 | 0.99 (0.95,1.03) | 0.61 |
| Colorectal | **1.04**^*^ **(1.01,1.06)** | **0.001** | **1.04**^*^  **(1.00,1.07)** | **0.03** | 1.00 (0.97,1.03) | 0.92 | 1.00 (0.96,1.05) | 0.91 |
| Liver | **1.03**^*^ **(1.01,1.05)** | **0.01** | 1.01 (0.98,1.04) | 0.70 | 1.03 (1.00,1.07) | 0.06 | 1.02 (0.97,1.08) | 0.45 |
| Gall bladder | 1.01 (0.96,1.06) | 0.81 | 1.02 (0.94,1.1) | 0.71 | 1.04 (0.99,1.10) | 0.14 | 1.02 (0.94,1.11) | 0.65 |
| Pancreas | 1.04 (1,1.09) | 0.07 | 1.04 (0.97,1.12) | 0.28 | 0.99 (0.94,1.05) | 0.84 | 1.02 (0.93,1.11) | 0.75 |
| Lung | 1.00 (0.98,1.02) | 0.98 | 0.98 (0.95,1.01) | 0.18 | 0.99 (0.96,1.02) | 0.49 | 0.98 (0.93,1.03) | 0.49 |
| Thyroid | 1.03 (0.98,1.07) | 0.24 | 1.06 (0.99,1.13) | 0.08 | 1.02 (1.00,1.04) | 0.10 | **1.04**^*^ **(1.01,1.08)** | **0.01** |
| Prostate | 0.99 (0.97,1.01) | 0.49 | 1.02 (0.99,1.06) | 0.24 |  |  |  |  |
| Kidney | 1.06 (0.99,1.12) | 0.08 | 1.10^*^ (1.00,1.21) | 0.05 |  |  |  |  |
| Bladder | 1.01 (0.97,1.06) | 0.59 | 1.07 (1.00,1.15) | 0.06 |  |  |  |  |
| Esophagus | 1.03 (0.97,1.1) | 0.31 | 1.02 (0.92,1.13) | 0.71 |  |  |  |  |
| Breast |  |  |  |  | 1.00 (0.98,1.03) | 0.81 | 0.99 (0.95,1.04) | 0.77 |
| Cervix uteri |  |  |  |  | 0.98 (0.93,1.04) | 0.58 | 0.98 (0.90,1.07) | 0.65 |
| Ovary |  |  |  |  | 1.02 (0.95,1.08) | 0.62 | 1.03 (0.94,1.13) | 0.53 |

HR, hazard ratio; CI, confidence interval; SBP, systolic blood pressure; DBP, diastolic blood pressure

The hazard ratio was estimated from the subjects without a history of diabetes and hypertension.

The hazard ratio was obtained while controlling for the age, income level, smoking, alcohol intake, and Charlson comorbidity Index score; ^*^ significant value, *P* < 0.05
